# Supplementary material for: Biophysical mechanism underlying compensatory preservation of neural synchrony over the adult lifespan
Source: Commun Biol. 2022 Jun 9;5:567. doi: 10.1038/s42003-022-03489-4 (PMC9184644; doi:10.1038/s42003-022-03489-4)
Supplement: Supplementary file 2 — Supplementary Information [file 42003_2022_3489_MOESM2_ESM.pdf]

SUPPLEMENTARY INFORMATION

Supplementary Note 1: Frequency suppression for different Noise Amplitudes and Natural Frequencies.

In order to establish the generality of our results, we repeated the analysis with different levels of noise inputs and by selecting different natural frequency bands. Just as in the main text, the metastability index, network frequency and phase locking value were estimated by varying conduction velocity(between 1-30m/s) and global scaling parameter(K). The parameter d, which scales noise amplitude, was set to 2,4. In the last row, we show parameter sweeps for the system when natural frequencies are scaled between $\omega_{min} = 6\,Hz$ and $\omega_{max} = 14\,Hz$. The third column demonstrates compensatory reduction of peak frequencies for different values of PLV.

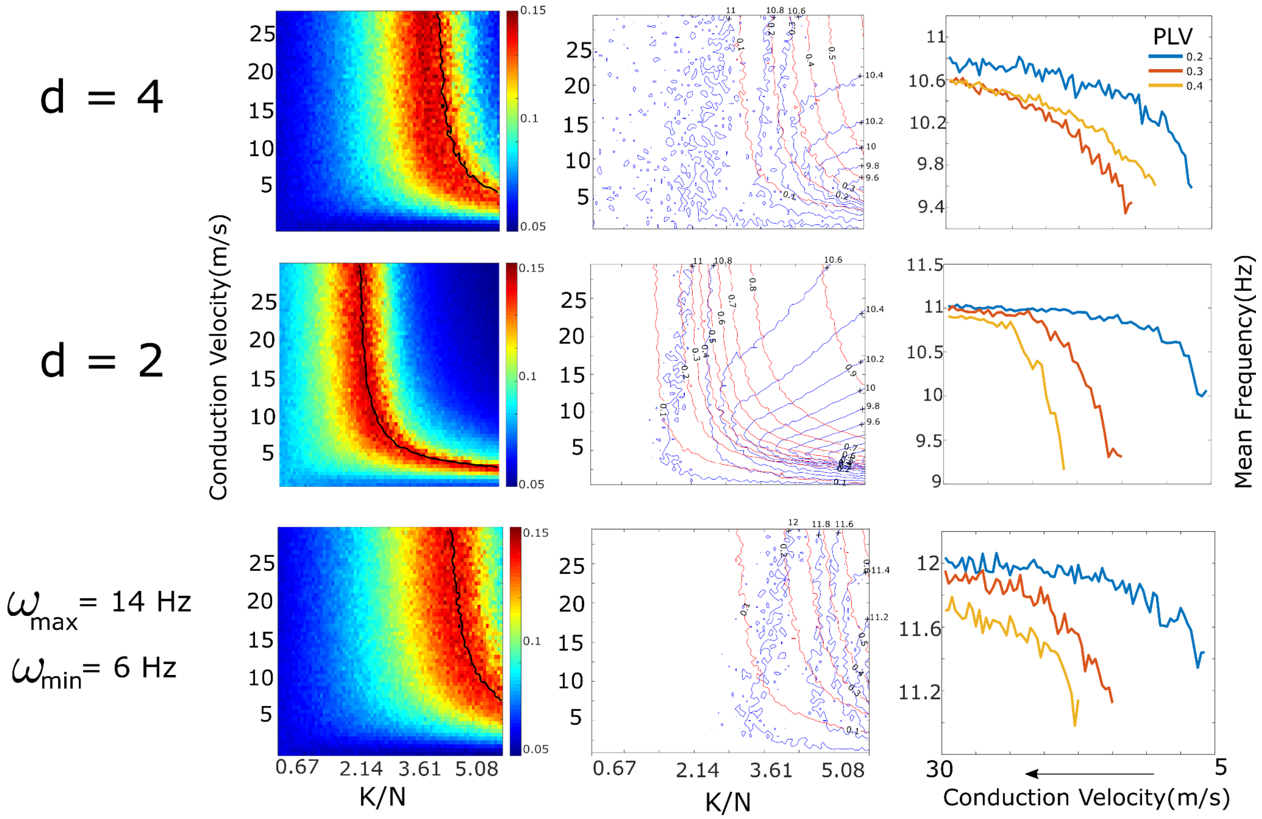

Supplementary Figure 1:Frequency suppression for different Noise Amplitudes and Natural Frequencies. Metastability (left column) , contour plots between peak frequency (blue) and phase locking (red) (middle column) and frequency vs conduction velocity curves (Right column) for varying noise levels (top 2 rows) and natural frequency ranges (bottom row).

Supplementary Note 2: Generalization of results across different datasets and parcellation schemas

We replicated our model findings on a separate DTI connectivity matrix as described in Cabral et. al(2014) [1]. The connectivity was parcellated according to the Automated Anatomical labelling(AAL) atlas(90 ROIs). Network frequency and phase locking were estimated by varying conduction velocity and global coupling. We observed qualitatively similar results with the AAL atlas. Network frequency reduces as the system of oscillators maintain phase locking at slower conduction velocities.

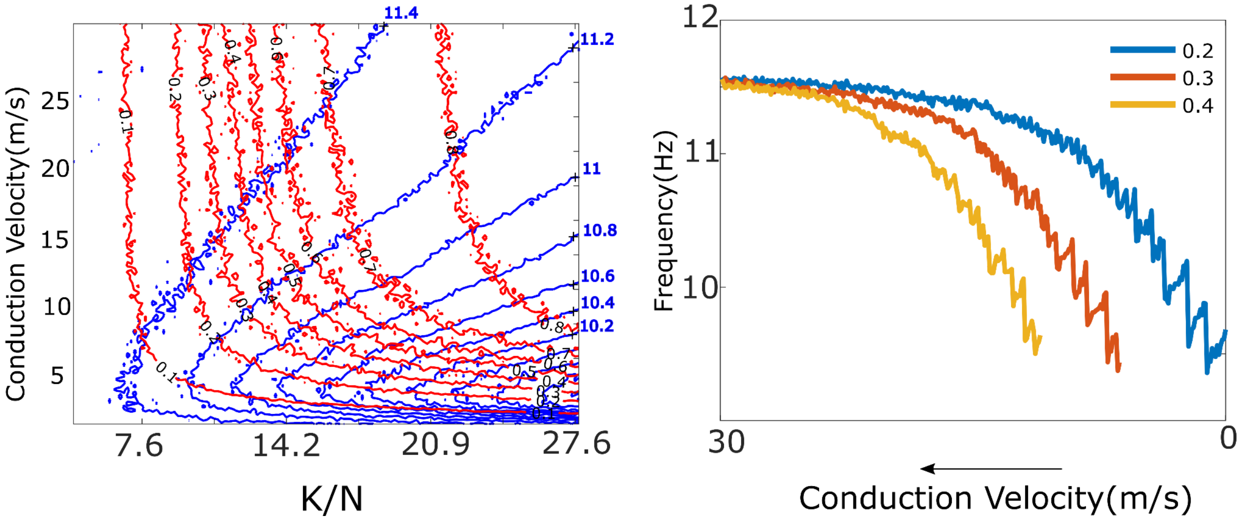

Supplementary Figure 2: Model generalization. Contour plot (left) between network frequency (blue) and phase locking (red) and Frequency vs conduction velocity plot (right) for AAL atlas.

Supplementary Note 3: Sensor Space analysis

We repeated our source-level analysis on MEG sensor level data. Participants possessing a distinct alpha peak in at least half of the magnetometers(N=102) were selected for PLI analysis(587 out of 650 participants).

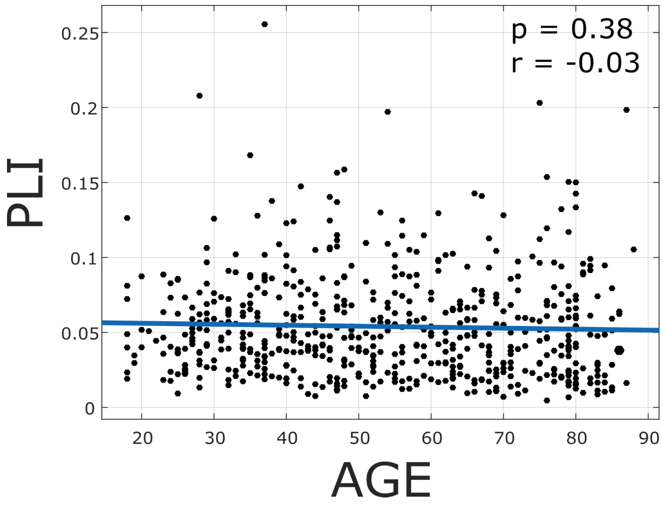

Supplementary Figure 3: Sensor space analysis, **Correlation between PLI and age in the sensor space (N = 587 subjects). Blue indicates the linear regression trendline.**

Supplementary Note 4: Deriving the macroscopic order parameter for the case of no delays

The phase dynamics are described by the following equation(**1**)-

$$\dot{\theta}_i = \omega_i + \frac{K}{N}\sum_{j=1}^{N}\sin(\theta_j - \theta_i)$$

The RHS may be written as(**2**)-

$$\frac{1}{N}\sum_{j=1}^{N}\sin(\theta_j - \theta_i) = Im[ze^{-i\theta_i}]$$

We can define a distribution function such that(**3**)-

$$\int_0^{2\pi} f(\omega,\theta,t)d\theta = g(\omega)$$

The distribution function tracks the evolution of the system of oscillators(**4**)-

$$f(\omega, \theta, 0) \to f(\omega, \theta, t)$$

Since the number of oscillators in the system remains unchanged, the distribution function must obey the following continuity equation(**5**)-

$$\frac{\partial f}{\partial t} + \frac{\partial \dot{\theta} f}{\partial \theta} = 0$$

This implies(6)-

$$\frac{\partial f}{\partial t} + \frac{\partial}{\partial \theta}[(\omega + \frac{K}{2}(ze^{-i\theta} - \widetilde{z}e^{i\theta}))f] = 0$$

Since f is a periodic function in θ, it may be written as a Fourier series(**7,8**)-

$$f(\theta, \omega, t) = f(\theta + 2\pi, \omega, t)$$

$$f = \frac{g(\omega)}{2\pi}(1 + \sum_{1}^{+\infty} f_n(\omega, t)e^{in\theta} + \sum_{-\infty}^{-1} \widetilde{f}_n(\omega, t)e^{-in\theta})$$

Ott and Antonsen propose the following ansatz(**9**)-

$$f_n(\omega, t) = \alpha^n(\omega, t)$$

Subject to the condition(**10**)-

$$|\alpha(\omega, t)| < 1$$

Inserting equations **9,10** in **7** yields the following(**11**)-

$$\frac{\partial \alpha}{\partial t} + \frac{K}{2}(z\alpha^2 - \widetilde{z}) + i\omega\alpha = 0$$

Inserting 9,10 into 4 yields(**12**)-

$$\widetilde{z} = \int_{-\infty}^{\infty} \alpha(\omega, t) g(\omega) dw$$

12 may be integrated using the residual theorem by making the substitution(**13**)-

$$z = \alpha^*(\mu - i\gamma, t)$$

Substituting 13 in 11 gives(**14**)-

$$\dot{z} = (i\mu - \gamma)z - \frac{K}{2}(z^2\widetilde{z} - z)$$

For more information about the derivation please refer [2].

.

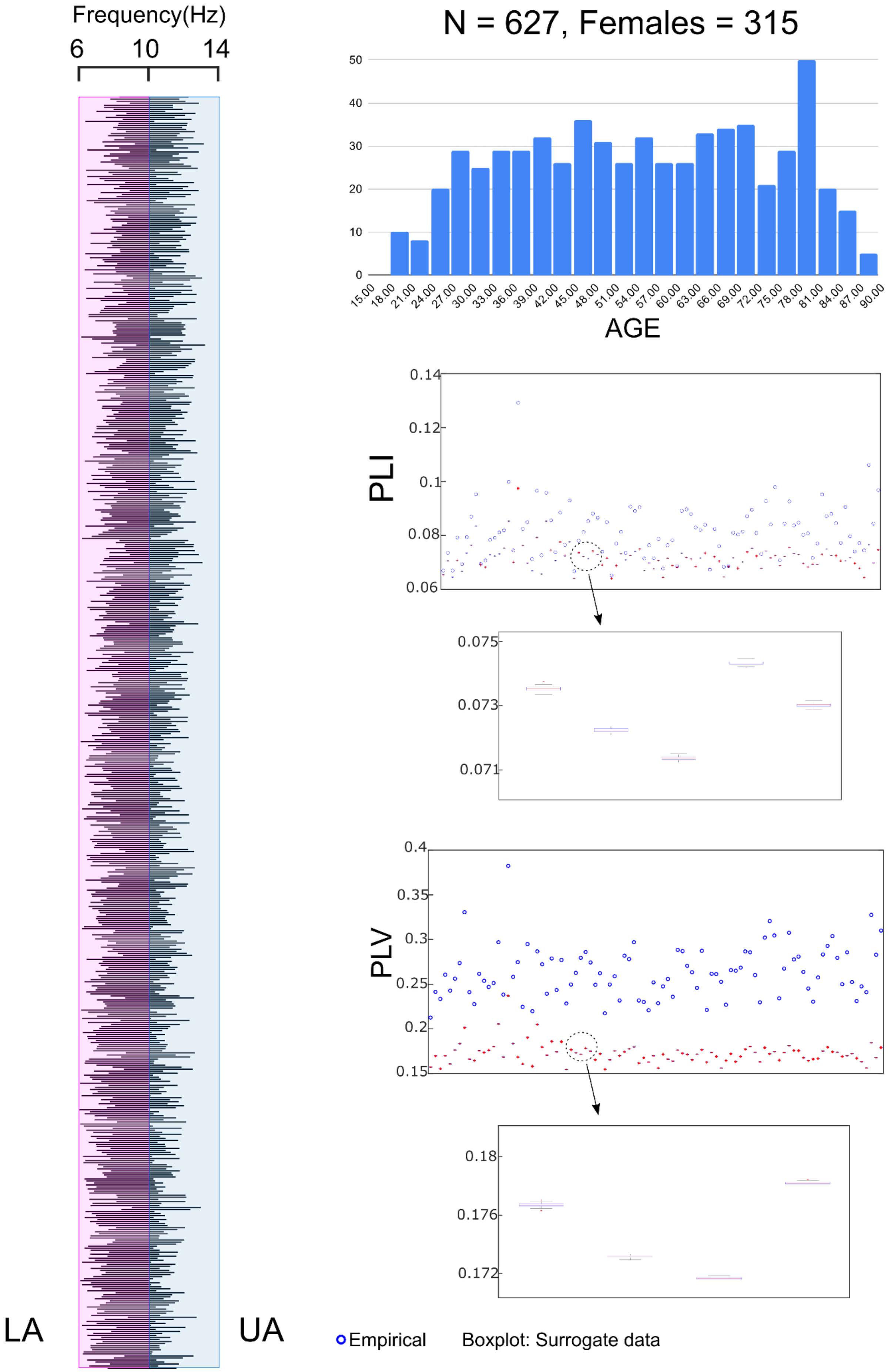
Frequency(Hz)
6
10
14
LA
UA
N = 627, Females = 315
50
40
30
20
10
0
15.00 18.00 21.00 24.00 27.00 30.00 33.00 36.00 39.00 42.00 45.00 48.00 51.00 54.00 57.00 60.00 63.00 66.00 69.00 72.00 75.00 78.00 81.00 84.00 87.00 90.00
AGE
PLI
0.14
0.12
0.1
0.08
0.06
0.075
0.073
0.071
PLV
0.4
0.35
0.3
0.25
0.2
0.15
0.18
0.176
0.172
Empirical
Boxplot: Surrogate data

Supplementary Figure 4: Participant Information and Bootstrapping . The distribution of LA, UA and SSA bands for all 627 participants (left column). Histogram- Age distribution for the 627 participants used in this study. (Bottom Right) 100 participants were chosen at random for permutation testing. PLV and PLI were calculated for the random permutation of epochs and averaged across ROI pairs. This was done for 100 iterations and significance was gauged from the resulting distribution. The box plot represents the distribution of surrogate data. On each box, the central mark indicates the median, and the bottom and top edges of the box indicate the 25th and 75th percentiles, respectively

Supplementary Note 5: Results from replicating Scally et. al.

A publicly available EEG dataset from N = 111 participants between the ages of 17-71 was used to replicate the findings of Scally et. al. Data was downloaded from the free data sharing format OpenNeuro (https://openneuro.org/datasets/ds003775/versions/1 .0.0). In agreement with our findings on the preservation of phase-locking by frequency slowing, Scally et. al.(2018) [3] report the absence of group differences in Phase locking at the peak alpha frequency between young and old participants. Following Scally et. al., we estimate phase locking between EEG electrodes and estimate the frequency of maximum phase locking in the alpha band for each subject and correlate it with PAF. We find a positive correlation between PAF and peak PLI frequency, indicating a relationship between spectral power and network synchrony

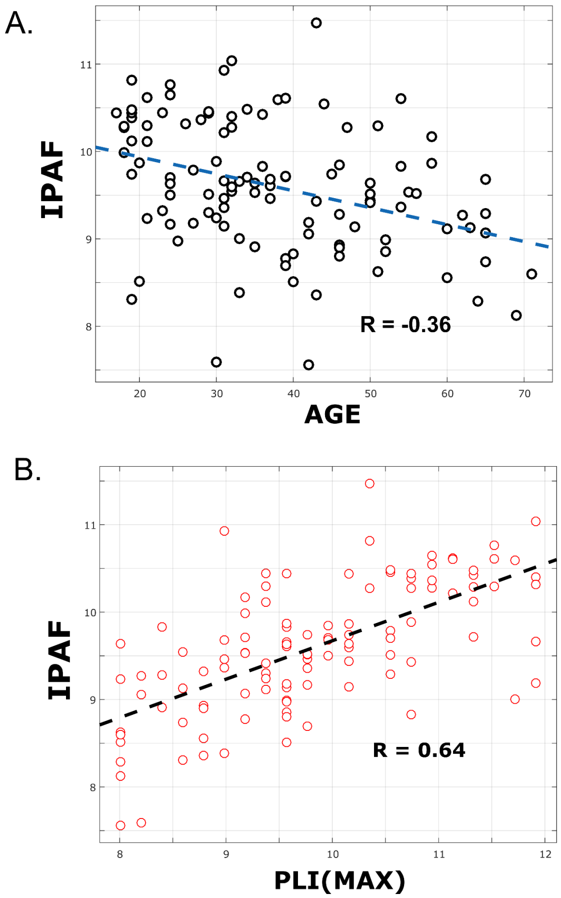

<u>Supplementary Figure 5: Scally Replication</u> A. IPAF as a function of age B. IPAF vs Frequency corresponding to maximum PLI. Dotted lines represent trendlines.

<u>Supplementary References:</u>

[1] Cabral, Joana, Henry Luckhoo, Mark Woolrich, Morten Joensson, Hamid Mohseni, Adam Baker, Morten L. Kringelbach, and Gustavo Deco. "Exploring mechanisms of spontaneous functional connectivity in MEG: how delayed network interactions lead to structured amplitude envelopes of band-pass filtered oscillations." *Neuroimage* 90 (2014): 423-435.

[2] Ott, Edward, and Thomas M. Antonsen. "Low dimensional behavior of large systems of globally coupled oscillators." *Chaos: An Interdisciplinary Journal of Nonlinear Science* 18, no. 3 (2008): 037113.

[3] Scally, Brian, Melanie Rose Burke, David Bunce, and Jean-Francois Delvenne. "Resting-state EEG power and connectivity are associated with alpha peak frequency slowing in healthy aging." *Neurobiology of aging* 71 (2018): 149-155.
