# Supplementary material for: Biophysical mechanism underlying compensatory preservation of neural synchrony over the adult lifespan
Source: Commun Biol. 2022 Jun 9;5:567. doi: 10.1038/s42003-022-03489-4 (PMC9184644; doi:10.1038/s42003-022-03489-4)
Supplement: Supplementary file 3 — Description Of Additional Supplementary Files [file 42003_2022_3489_MOESM3_ESM.pdf]

Description of Additional Supplementary Files

File name: Supplementary Data 1
Description:

Data used for plotting Figure 2 and 4.

PLI and PLV for LA, SSA and UA band (Fig 2).

Natural Frequency,metastability values, conduction delay distribution for 5,10,15,20 m/s and PAF and PLV obtained from simulations (Fig 4).
